# Safety margin in irradiation of colorectal liver metastases: assessment of the control dose of micrometastases

**DOI:** 10.1186/1748-717X-5-24

**Published:** 2010-03-24

**Authors:** Max Seidensticker, Peter Wust, Ricarda Rühl, Konrad Mohnike, Maciej Pech, Gero Wieners, Günther Gademann, Jens Ricke

**Affiliations:** 1 Klinik für Radiologie und Nuklearmedizin, Universitätsklinikum Magdeburg, Otto-von-Guericke-Universität Magdeburg, Germany; 2 Klinik für Strahlenheilkunde, Charité Universitätsmedizin Berlin, Campus Virchow Klinikum, Germany; 3 Klinik für Strahlentherapie, Universitätsklinikum Magdeburg, Otto-von-Guericke-Universität Magdeburg, Germany

## Abstract

**Backround:**

Micrometastases of colorectal liver metastases are present in up to 50% of lesions. In this study we sought to determine the threshold dose for local control of occult micrometastases in patients undergoing CT (computed tomography)-guided brachytherapy of colorectal liver metastases.

**Materials and methods:**

Nineteen patients demonstrated 34 local tumor recurrences originating from micrometastases after CT-guided brachytherapy of 27 colorectal liver metastases. We considered a local tumor recurrence as originating from a micrometastasis if tumor regrowth occurred adjacent to a formerly irradiated lesion and the distance of the 3D isocenter of the new lesion was ≤ 23.5 mm from the previous tumor margin. Follow-up MRI was fused with the planning-CT and dosimetry data. Two reviewers independently indicated the dose exposure at the isocenter of the micrometastases. Statistical analysis included an analysis of variance (ANOVA) using backward selection. 95% tolerance intervals with coverage of 87.5 and 75% of the data of the normal distribution were calculated.

**Results:**

The median distance of the micrometastases to the margin of the originating colorectal metastases was 8.75 mm (1-21 mm). Dose exposure at the isocenter was 12.25 Gy (7-19.8) in median. We stratified according to the distance from the isocenter to the initial tumor margin: ≤ 9 mm, > 9-15 mm and > 15 mm. The median dose in the according isocenters was 13.18, 11.6 and 11.85 Gy. The threshold dose failing to prevent micrometastasis growth was sigificantly higher in a subgroup of lesions with ≤ 9 mm distance as compared to > 15 mm (13.18 vs 11.85 Gy). Adjuvant chemotherapy correlated with greater distance of micrometastasis growth to the tumor but not with the threshold dose.

**Conclusion:**

To prevent loss of local tumor control by continuous growth of micrometastases a threshold dose of 15,4 Gy (single fraction) should be delivered at a distance of 21 mm to the gross tumor margin.

## Backround

For the treatment of liver metastases from colorectal carcinoma, surgery as well as percutaneous image guided tumor ablation have demonstrated favourable results with respect to an improvement of the patient's prognosis [1-7]. Both the surgical as well as the minimal, or, in case of percutaneous irradiation, non-invasive approach require a safety margin around the target to reduce the risk of a recurrence and to gain a better prognosis [1,8-12]. Recent publications have drawn attention to the presence of radiologically invisible micrometastases or microsatellites, respectively (in the following we apply the term micrometastases). These micrometastases directly originate from and are found frequently adjacent to colorectal liver metastases [12-16].

Occult tumor cell nests such as micrometastases play a significant role in recurrent tumor growth after local tumor treatments. A histopathologic study of 31 liver specimen after liver resection of colorectal metastases demonstrated micrometastases deriving from neighbouring macrometastases in 56% of the cases. The mean distance between micrometastasis and originating macrometastases was 7.5 mm (SD (standard deviation) 8 mm) [13]. Hence, treatment planning in liver metastases irradiation must not only consider the radiologically visible tumor bulk, but also the extension of subclinical disease around the gross tumor. Radiobiologically, local control of low cell densities is required. The according dose will be lower than control doses for gross tumor volumes [17,18]. Considering the distance subclinical micrometastases may have from the gross tumor volume, knowledge about the control dose for micrometastases helps to reduce the clinical target volume specifically in irradiation techniques with steep dose gradients.

In the study described herein we retrospectively analyzed recurrent tumor growth after CT-guided brachytherapy of colorectal liver metastases. We included only patients displaying tumor recurrences identified as originating from micrometastases around the initial target lesion. The aim of this study was to determine the threshold dose for local control of micrometastases of colorectal liver metastases.

## Materials and methods

### Patient identification

We included 19 patients (female, n_patients _= 8; male, n_patients _= 11) with a mean age of 64 years (range 49-86 years). All patients displayed nodular tumor regrowth (n_lesions _= 34) during follow up after CT-guided brachytherapy of 27 colorectal liver metastases. These tumor recurrences were classified as originating from micrometastases *(for definition of micrometastases see standard of reference)*. Primary tumor site was colon in 11 and rectum in 8 patients. After CT-guided brachytherapy, 4 patients had received chemotherapy (FOLFIRI (×1), irinotecan (×2), FU/FA (×1)) as adjuvant treatment. All other patients did not receive systemic treatment in the time interval between local treatment and confirmation of tumor regrowth.

### Standard of reference and definitions

**Figure 1:**
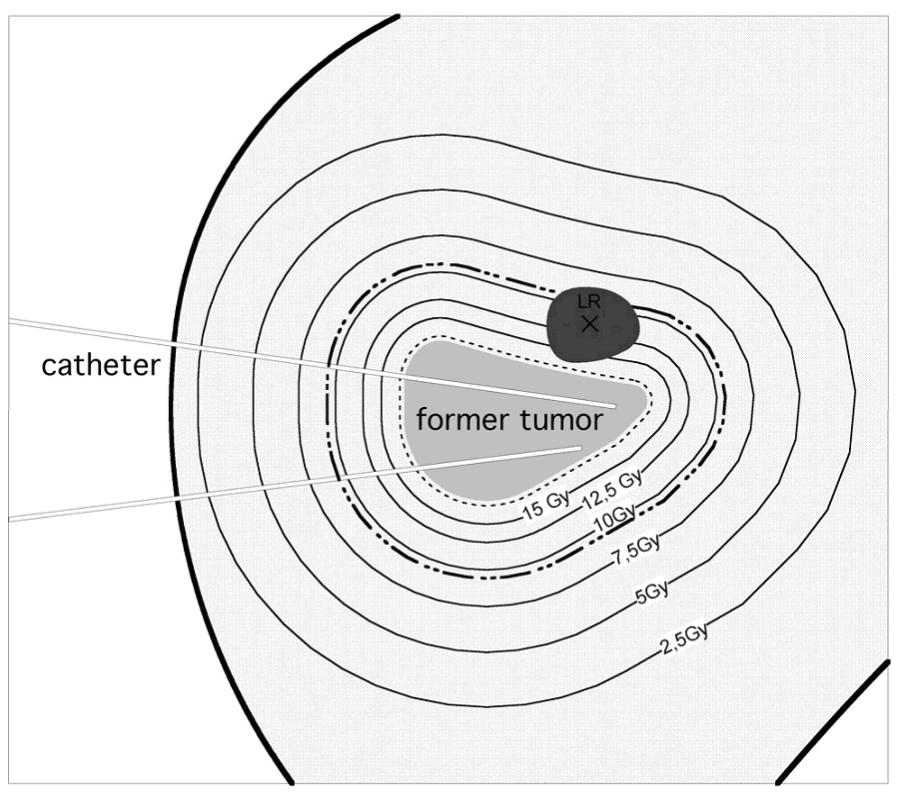
**Scheme of a follow-up MRI merged with the initial dosimetry displaying a tumor recurrence of a micrometastasis (LR)**. The black cross in LR marks the 3D isocenter. The dashed line describes the CTV around the colorectal liver metastasis which had been treated initially. The bold dashed line outlines 23.5 mm distance from the initial tumor margin.

Colorectal liver metastases were confirmed by histopathology prior to the initial CT guided brachytherapy. Tumor burden prior to therapy was assessed by MRI (magnetic resonance imaging) based volumetry. Diagnosis of local tumor recurrence during follow up was confirmed by tumor growth in contrast enhanced MRI. No biopsy was taken from these tumor recurrences. We considered a local tumor recurrence to be originating from a micrometastasis if all of the following applied:

a) the new lesion occurred adjacent to a previously treated lesion.

b) the new lesion had a nodular shape applying a asymmetrical appearance in conjunction with the original, pretreated lesion.

c) The 3D isocenter of the new lesion was ≤ 23.5 mm from the initial margin of the metastasis before brachytherapy (adapted from histopathological studies by Nanko et al [13]) (figure 1).

### Eligibility criteria

We excluded patients presenting a symmetric tumor regrowth of the irradiated metastasis or patients with disseminated new intrahepatic tumor deposits.

### Interventional technique CT-guided brachytherapy

The technique of CT-guided brachytherapy has been described elsewhere [19]. The placement of the brachytherapy applicators was performed at a Fluoroscopy CT (Siemens, Erlangen, Germany). For treatment planning purposes, a spiral CT of the liver (slice thickness: 5 mm; increment: 5 mm) enhanced by intravenous administration of iodide contrast media (100 ml Ultravist 370, flow: 3 ml/s; start delay: 80 s) was acquired using breathhold technique after positioning of the brachytherapy catheters in the tumor.

Depending on tumor geometry and lesion size, a median of 4 catheters was used in our patients (range: 2 - 20 catheters).

The 3D CT data set acquired after catheter positioning was transferred to the treatment planning unit (BrachyVision^®^, Varian Medical Systems, Charlottesville, VA, USA).

A Radiooncologist defined the CTV (clinical target volume) including a safety margin of 2 mm in the 3D CT data. Threshold doses for local control of colorectal liver metastases using this approach have been published recently [5]. No image fusion of MRI pre treatment with planning CT was performed since in all patients tumor conspicuity on CT was sufficient for treatment planning. The prescribed and applied minimal dose inside the CTV was 15 Gy at median (12 to 25 Gy).

The high dose rate afterloading system employed a ^192^Iridium source of 10 Ci (Gammamed^®^, Varian Medical Systems, Charlottesville, VA, USA). The source diameter was < 1 mm. Dwell positions were located every 5 mm. Dwell times were corrected automatically according to the actual source strength. The true mean duration of the irradiation was 2018 seconds (range: 1088 to 4666 seconds). Normalized to 10 Ci according to the actual source strength the theoretical duration would have been 1633 seconds (range: 639 to 3825 seconds). A single dose rate can not be calculated due to variable catheter geometries and differing distances of tumor tissue to the catheters. According to the irradiation time and the known minimal dose at the tumor margin a minimal dose rate can be calculated ranging from 11-84 Gy/h (mean 43).

### MRI Baseline and Follow-up

All patients underwent MRI (Gyroscan NT 1.5T, Philips, Best, The Netherlands) of the liver 1 day prior to brachytherapy and in follow up 6 weeks and every 3 months after treatment. The MRI protocol consisted of the following sequences: T2-w UTSE (T2-weighted ultrafast spinecho) (TE/TR (time to echo/timo to repetition) 90/2100 ms) with and without fat suppression, T1-w GRE (T1-weighted gradient recalled echo) (TE/TR 5/30 ms, flip angle 30°) pre-contrast, 20 s post intravenous administration of 15 ml Gd-BOPTA (Gadobenate dimeglumine, Multihance^®^, Bracco, Princeton, USA), and 2 h post injection of intravenous Gd-BOPTA. The slice thickness was 5 mm (T1-w sequences) and 8 mm (T2-w sequences) acquired in interleaved mode with no gap applied.

### Tumor assessment and image registration

**Figure 2:**
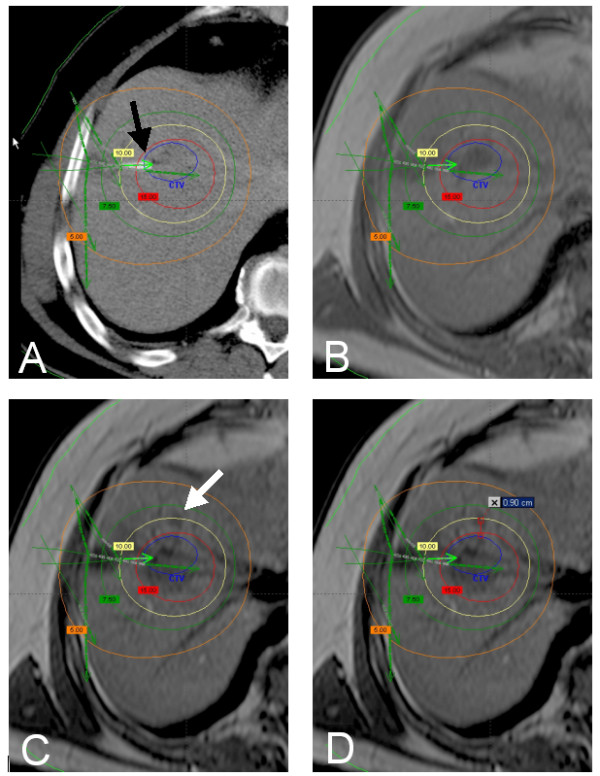
**A: planning CT with overlayed dosimetry (BrachyVision^®^) showing a colorectal liver metastasis in segment 8**. One catheter tip is displayed directly (black arrow), more catheters in other levels of the liver are indicated by green arrows. Verification of correct definition of the CTV was performed by image fusion of the planning CT with a MR scan (T1 GRE without contrast media) obtained 3 days prior to treatment (B). Local recurrence (white arrow) 6 months after treatment (MR, T1 GRE without contrast media, C). The distance of the 3D isocenter of the local recurrence from the initial tumor margin is 9 mm. Thus, the local tumor recurrence meets the criteria for micrometastasis growth (D). The dose initially applied in the center of the micrometastasis was 10.9 Gy.

Plain T1-w GRE sequences were used to determine the location and the size of nodular local tumor recurrences [20]. Image fusion of the MRI sequence showing the regrowth of the micrometastases with the former treatment plan was performed by BrachyVision^®^. The algorithm employs a rigid local semi-automated point based 3D-3D image registration. Match points were defined on corresponding landmarks such as branches of the portal vein to enable fusion of MR and planning CT/dosimetry data. Landmarks were restricted to the liver and chosen as close to the lesion as possible, i.e. limited to the identical liver lobe. As a result of this procedure, BrachyVision^® ^simultaneously displayed the treatment plan as well as the anatomical structures of the MRI with a maximum deviation of < 5 mm (figure 2).

One radiologist and one radiooncologist (reader 1 and 2) evaluated the combined MRI/dosimetry data independently. The reviewers individually calculated the largest diameter of the recurrent tumor mass, its 3D-isocenter coordinates ("center of the recurrent mass") as well as the dose at this respective point. In addition, they measured the distance of the 3D isocenter to the initial tumor margin prior to the first brachytherapy. By image fusion of MRI (T1-w GRE pre contrast) 1 day prior to treatment with follow up MRI visible tumor as origin of the recurrent tumor mass could be excluded.

### Statistical analysis

Results of continuous data are displayed as medians and ranges, results of frequency data as counts and percentages. For the analysis, independence between lesions within the same patient was assumed as the treatment was applied locally and not systemic, so that the treatment of one lesion did not affect a second lesion and any micrometastasis with this second lesion.

The agreement between the two readers evaluating the applied dose was measured by the intra-class correlation coefficient based on a linear model.

For two-group comparisons of the medians two-sided Wilcoxon rank sum tests were used. Measured doses were assumed to be normally distributed. Therefore t-Tests were used to test for pairwise differences in doses. We used a mixed linear model to account for the repeated measurements of doses for each lesion by the two readers. Independence was used as working correlation matrix.

Important independent factors to explain the variation of the measured dose in the center of the recurrent mass were evaluated by an analysis of variance (ANOVA) using backward selection to select significant factors. Based on the final model, 97.5% upper tolerance limits with coverage of 87.5% and 75% were calculated. The maximum upper tolerance limit (incuding 87.5% or 75% of the data, respectively) for all combinations of significant factors were used to define the "insufficient doses to prevent micrometastasis growth". The tolerance intervals were extrapolated to a maximum distance of 23 mm from the limit of the primary lesion as the data only contained data up to 21 mm. p-values below 0.05 were regarded as statistically significant.

Calculations were performed using R software (version 2.7.1, R Development Core Team (2008)) and SAS^® ^9.2 (SAS-Institute, Cary, NY).

## Results

The mean diameter of the colorectal metastases treated by CT-guided brachytherapy was 4.5 cm (range 1.5-11 cm), the volume 50 ccm (range 3-630 ccm). The shape of the respective metastases was oligonodular (asymmetric confluent) versus round (regular spheroid) in 32% and 68% of lesions, respectively. The minimal dose at the tumor margin applied during CT-guided brachytherapy was 15 Gy (range 12-25 Gy). The activity factor of the ^192^Iridium source was 1.17 (range 0.97-1.83).

Recurrent tumor categorized as micrometastasis growth was depicted at a mean follow up of 6 months (range 3-22 months) with 88% of all lesions occuring within 12 months.

Local tumor recurrences from micrometastases displayed a mean axial diameter of 1.5 cm (range 0.8-2.4 cm), the mean tumor volume was 1.76 ccm (0.27-7.23 ccm).

The distance of the 3D isocenter of the micrometastases to the margin of the originating colorectal metastases was 8.75 mm (range 1-21 mm, Q25: 3 mm, Q75: 15 mm).

**Figure 3:**
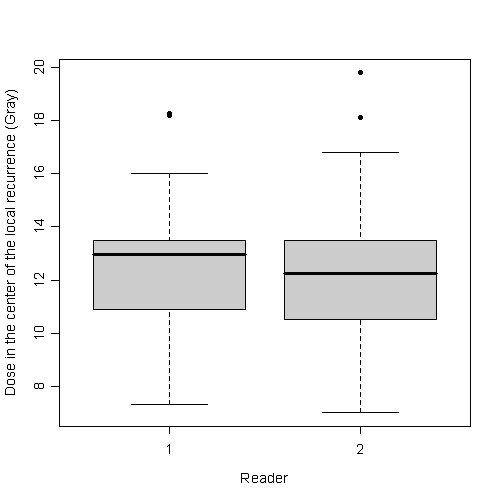
**Boxplot of the point dose at the center of each micrometastasis as indicated by both readers**.

The dose in the 3D-isocenter of the micrometastases was 12.95 Gy (Reader 1: 7.33-18.75 Gy, Q25: 10.93 Gy, Q75: 13.47 Gy) and 12.25 Gy (Reader 2: 7-19.8 Gy, Q25: 10.5 Gy, Q75: 13.5 Gy) (figure 3).

**Figure 4:**
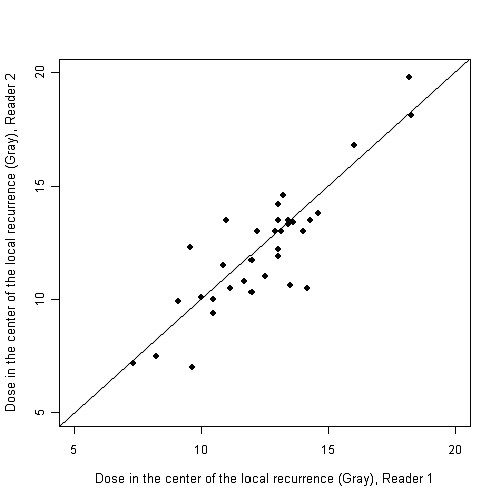
**Intra-class correlation for comparison of the readers demonstrating a very high interobserver correlation (0.86)**.

The interobserver-correlation was 0.86 (figure 4). Since the interobserver-correlation yielded this very high agreement, a cumulative evaluation was performed during further analyses.

**Figure 5:**
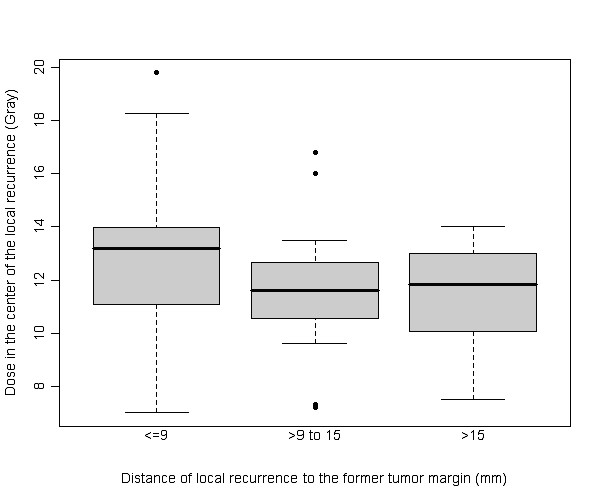
**Boxplot of the dose at the 3D isocenter of each micrometastasis grouped according to the distance to the margin of the originating metastases across readers**. The difference between the doses measured in the group ≤ 9 mm (in median 13.18 Gy) and > 15 mm (in median 11.85 Gy) was significant (p = 0.0442).

We stratified tumor recurrences from micrometastases according to the distance from the 3D-isocenter to the initial tumor margin: ≤ 9 mm (n = 18), > 9-15 mm (n = 8) and > 15 mm (n = 8). The median dose across readers in the according isocenters was 13.18 Gy, 11.6 Gy and 11.85 Gy, respectively (figure 5). Significant pairwise differences between the groups were only found for distances ≤ 9 mm as compared to > 15 mm for the assessments across readers (p = 0.0442).

**Figure 6:**
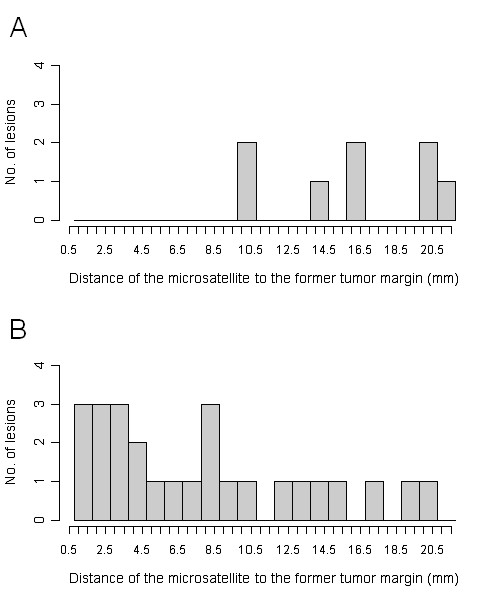
**Distance of the micrometastases to the former tumor margin stratified by history of adjuvant chemotherapy (yes: figure 6 A, no: figure 6 B), whereas tumor growth occurred in significantly greater distance from the originating metastasis when adjuvant chemotherapy was applied (p = 0.0038)—** r**elated lower dose levels failed to reach statistical significance (p > 0.05).**

Stratification of the tumor recurrence from micrometastases according to a history of adjuvant chemotherapy (yes/no) after initial irradiation showed a significantly higher distance of the 3D-isocenter to the originating metastases when adjuvant chemotherapy was applied (p = 0.0038) (figure 6). However, despite the influence of adjuvant chemotherapy regarding the distance of the isocenter, lower dose levels at greater distances as a result of the dose gradient failed to reach significance (p > 0.05).

Results of the ANOVA analysis are displayed in table 1. Upper 97.5% tolerance limits were calculated with coverage of 87.5% and 75% of the data. In essence, doses indicated refer to the threshold doses avoiding tumor growth from micrometastases in 87.5% or 75% of the cases. The Maximum upper 97.5% tolerance limit with coverage of 87.5% for the distance of 21 was 15.4 Gy: 87.5% of the doses in the isocenters of the micrometastases with a distance of < 21 mm to the initial tumor margin were less than 15.4 Gy. Thus < 15.4 Gy at a distance of < 21 mm was insufficient to avoid tumor growth from micrometastases in 87.5% of the cases.

**Table 1:** Results of the ANOVA analysis

Distance of the local recurrence to the former tumor margin (mm)	Maximum upper 97.5% tolerance limit with coverage of 87.5% (Gy)	Maximum upper 97.5% tolerance limit with coverage of 75% (Gy)
1	18.66	17.56

2	18.49	17.40

3	18.32	17.23

4	18.16	17.06

5	17.99	16.90

6	17.82	16.73

7	17.66	16.57

8	17.49	16.41

9	17.33	16.24

10	17.17	16.08

11	17.01	15.92

12	16.85	15.76

13	16.69	15.59

14	16.53	15.43

15	16.37	15.27

16	16.22	15.11

17	16.06	14.96

18	15.90	14.80

19	15.75	14.64

20	15.60	14.48

21	15.45	14.33

22	15.30	14.17

23	15.15	14.01

Maximum upper 97.5% tolerance limits with coverage of 87.5% and 75%. 87.5% means that 87.5% of the data (dose measured in the center of the micrometastasis) are below the upper limit of the tolerance interval (with a confidence of 97.5%).

The Maximum upper 97.5% tolerance limit with coverage of 87.5% for the distance of 21 mm was 15.4 Gy: 87.5% of the doses in the isocenters of the micrometastases with a distance of < 21 mm to the initial tumor margin were less than 15.4 Gy. Thus < 15.4 Gy at a distance of < 21 mm was insufficient to avoid tumor growth from micrometastases in 87.5% of the cases.

As independent factors the distance between the isocenter and the initial tumor margin (the higher the distance the lower the dose, p = 0.0004) as well as the geometry of the initial liver lesion (oligonodular shape was associated with a higher threshold dose, p = 0.009) significantly influenced the threshold doses for micrometastasis growth. The size of the irradiated colorectal liver metastases showed no influence on the threshold dose.

## Discussion

In surgical and local treatment of colorectal liver metastases the margin status is consistently related to prognosis after treatment. Numerous authors have investigated the significance of margin status for resection of colorectal liver metastases [1,8-12]. Although the existence of positive margins is shown to account for a high rate of local recurrences, practical guidelines for the extent of a safety margin are not fully understood. Surgical studies dedicated to this issue have demonstrated a lower rate of local tumor recurrences in patients resected with a safety margin > 1 cm margin [9,12]. In a study of Wray et al. in 112 patients undergoing liver resection with a safety margin < 1 cm 45% developed a local tumor recurrence [9].

These clinical observations are supported by histopathological findings. Previous authors have described a direct invasion of cancer cells into bile duct and lymphatic vessels inducing satellite lesions in close distance [12,13]. The frequency of such lesions termed micrometastases or microsatellites is influenced by distance to the macrometastases, presence of a pseudocapsule, lymphocyte infiltration separating metastases and neighbouring liver parenchyma, and the morphologic type of the lesion (round vs. oligonodular) [13,14,21,22]. Histopathologically, micrometastases were depicted more often with the confluent nodular (oligonodular) morphology [22]. A recent study has proven a negative impact of oligonodular lesion shape on local progression free survival in colorectal cancer patients undergoing irradiation therapy (CT- guided brachytherapy) of liver metastases [5]. These findings suggest that the presence of micrometastases frequently found in oliginodular lesions may at least in part be responsible for early local failures.

Hence, the presence of radiologically occult micrometastases around colorectal liver metastases has to be considered when delineating the clinical target volume for local irradiation. Nanko et al. [13] described the mean distance of micrometastases to the margin of the radiologically visible macrometastases of 7.5 mm ± 8 mm. An explanation for this high standard deviation was not stated by the authors; however, a low number of micrometatastases at a larger distance to the initial tumor margin might have been causative. Assuming an underlying Gaussian distribution 95% of the micrometastases were found in a distance of < 23,5 mm. This calculation led to our definition of micrometastasis regrowth, with asymmetrical, nodular growth at a total distance of ≤ 23.5 mm from the initial tumor margin after brachytherapy. In our study, the mean distance from the 3D isocenter of the micrometastases to the former tumor border was 9.6 mm ± 6.5 mm (median: 8,75 mm). This finding correlates closely with the histopathological data published by Nanko et al. of 7.5 mm ± 8 mm and it supports the validity of our definition of micrometastases [13].

Furthermore, both histopathology by Nanko et al. as well as our own data describe a higher rate of micrometastases in close proximity to the tumor margin (74 and 53% ≤ 9 mm, respectively) [13]. In addition, the cell density in these nearby lesions has been described to be higher than at greater distance [13,16]. Wakai et al described a tenfold higher cell densitiy of the micrometastases in the close zone of ≤ 10 mm around the tumor compared to the distant zone > 10 mm [16]. Radiobiologically, a higher radiation dose is needed to achieve complete cell kill in areas of higher tumor cell density [17,18]. The histopathological proof of higher cell density of micrometastases at close proximity may well explain our own finding that micrometastases located nearby the macrometastases occurred despite marginally increased doses (table 1 and figure 5).

The inherent advantage of computed tomographic guidance for interstitial irradiation of liver malignancies is the accuracy of the dose administration whereas external beam liver radiotherapy is hampered by a discrepancy between planned and radiated target, mainly due to breathing movements of the organ (up to 10 mm in craniocaudal direction) [23,24]. Therefore, the PTV in external beam liver radiotherapy exceeds the CTV substantially [3]. In CT-guided brachytherapy the catheters are positioned and fixed inside the tumor. Hence, organ motion is not a limiting factor and the CTV and PTV are theoretically not different. An implementation of the gained data regarding the threshold dose of micrometastases in treatment planning of CT guided brachytherapy of colorectal liver metastases seems feasible whereas in external beam liver radiotherapy an additional extension of the radiated field will cumber at least the therapy of big metastases.

With respect to the methodology used, some aspects need to be discussed. First, although performing a locally focused 3D-3D registration of the liver CT and MRI the deviation was up to 5 mm. This mismatch in image registration of CT and MRI of the liver is in good congruence to other studies [25,26]. Due to different modalities and possible organ distortion between the image studies a small registration error is not avoidable. The direction of registration mismatch is variable and not systemetical, thus we do believe that the margin as calculated by us accounts for this deviation.

Second, our determination of the 3D isocenter of a micrometastasis as its primary location was based on the assumption of centrifugal tumor growth [27-30]. Simulated three-dimensional tumor growth dynamics of brain tumors by Kansal et al. revealed spherical growth even if multiple cell strains participated in growth [31].

Third, statistical analysis by ANOVA was used to determine the threshold doses failing to prevent micrometastasis growth after brachytherapy. In consequence, our assumptions are limited to the negative proof in lesions displaying treatment failure. The positive affirmation, i.e. the dose assuring micrometastasis control could not be tested since micrometastases were occult at the time of the initial treatment. However, the consistency of the data drawn from the negative proof in this study is extremely high. As can be seen in table 1, an increasing distance of the isocenter of the micrometastases from the originating metastases corresponds to a quite discrete linear dose decline for both the 87.5% and the 75% interval. Stratification in micrometastases at a distance of < 9 mm, 9-15 mm and > 15 mm revealed significance for the threshold dose only for nearby lesions compared to the very distanced lesions, a phenomenon which we attribute to the decreasing cell density of remote micrometastases as has been proven by histopathology [13,16]. In contrast to this, in CT-guided brachytherapy the dose gradient outside the CTV typically shows a strong decline to approximately 25% of the dose at a distance of 2 cm [32]. We conclude that our results gained by employing the negative proof are statistically very consistent and thus demonstrated their validity for the determination of the threshold dose to prevent recurrent micrometastasis growth.

In summary, micrometastases are frequent in patients with colorectal liver metastases. According to histopathological results, micrometastases may be encountered in up to 50% of metastases with a predominance in lesions displaying an oligonodular shape. To prevent loss of local tumor control by continuous growth of micrometastases after single fractioned irradiation of colorectal liver metastases, we recommend to deliver a dose of at least 15,4 Gy at a distance of 21 mm to the gross tumor margin. Adjuvant chemotherapy had a positive impact on the development of tumor growth from micrometastases.

## Competing interests

The authors declare that they have no competing interests.

## Authors' contributions

MS participated in the study design and drafted the manuscript; PW participated in the design of the study; RR performed image fusion and screened patients for inclusion; KM performed image fusion and screened patients for inclusion; MP served as reader; GW performed image fusion and screened patients for inclusion; GG served as reader; JR conceived of the study and paticipated in its design and coordination. All authors read and approved the final manuscript.
